# Forest gaps slow the sequestration of soil organic matter: a humification experiment with six foliar litters in an alpine forest

**DOI:** 10.1038/srep19744

**Published:** 2016-01-21

**Authors:** Xiangyin Ni, Wanqin Yang, Bo Tan, Han Li, Jie He, Liya Xu, Fuzhong Wu

**Affiliations:** 1 Long-term Research Station of Alpine Forest Ecosystems, Key Laboratory of Ecological Forestry Engineering, Institute of Ecology and Forestry, Sichuan Agricultural University, Chengdu 611130, China

## Abstract

Humification of plant litter containing carbon and other nutrients greatly contributes to the buildup of soil organic matter, but this process can be altered by forest gap-induced environmental variations during the winter and growing seasons. We conducted a field litterbag experiment in an alpine forest on the eastern Tibetan Plateau from November 2012 to October 2014. Six dominant types of foliar litter were placed on the forest floor in various forest gap positions, including gap centre, canopy gap, expanded gap and closed canopy. Over two years of incubation, all foliar litters were substantially humified especially during the first winter, although the newly accumulated humic substances were young and could be decomposed further. The forest gaps exhibited significant effects on early litter humification, but the effects were regulated by sampling seasons and litter types. Compared with the litter under the closed canopy, humification was suppressed in the gap centre after two years of field incubation. The results presented here suggest that gap formation delays the accumulation of soil organic matter, and reduces soil carbon sequestration in these alpine forests.

Humification is the process by which plant litter is converted into soil organic matter (SOM) and is vital for improving soil fertility^1^,^2^. However, knowledge regarding the humification process of vascular plant litter remains unclear. The three-stage model of litter decomposition has been thoroughly demonstrated in recent years^3^, and the theory suggests that humus may be formed during the late stage of decomposition; however, we speculate that the decomposition of freshly shed litter may be accompanied by humification^4^,^5^. Furthermore, the accumulation of SOM in forested ecosystems not only depends on the amount of litter input but also on the degree of humification of the litter^6^. However, humification process is complex and is based on both biotic and abiotic pathways^2^. Theoretical analyses have suggested that humification may require microbial priming^7^ or is dependent on litter chemistry^8^. However, observations thus far have not been representative enough, and no general paradigm has been created to measure litter humification rate^9^. In recent decades, soil scientists have explored and attempted to characterize SOM by using heterogeneous analytical approaches from multidisciplinary perspectives^10^. These approaches included chemolytic^11^, spectroscopic^12^ and nuclear magnetic resonance (NMR) techniques^13^,^14^,^15^. However, these methods are disjointed and often produce conflicting results^9^. Measuring the colorimetric changes of absorptive spectra, which is recommended by Kumada *et al.*^16^, is a convenient method for evaluating humification^17^. This method indirectly assesses the structural changes that occur during humification and can provide some important information regarding SOM characteristics. In this case, ΔlogK (the logarithm of the absorbance at 400 and 600 nm) and E4/E6 (the ratio of the absorbance at 465 and 665 nm) represent the conjugate change of the aromatic carbon in litter, and lower values qualitatively indicate a higher degree of humification. Because litter is humified, cumulative humic substances can be classified based on their levels of maturity^16^.

Climate change will be exacerbated in frigid biomes, such as pristine alpine forests^18^. Our previous field investigation implied that climatic disasters, such as heavy snow or wind damage caused to tree canopies^19^, may accelerate the creation of forest gaps (i.e., canopy gap and expanded gap) in these high altitude ecosystems^20^. Increasing evidence indicates that forest gap-induced canopy openings allow more light to reach the surface litter, resulting in higher temperatures and higher moisture inputs^21^,^22^, which are responsible for chemical changes in the litter^23^,^24^. However, the environmental factors in forest gaps in pristine alpine forests vary between the winter and growing seasons. During the winter, the forest gaps are covered by deep snow, which acts as a thermal insulator^25^ and reduces the incidence of freeze-thaw cycles^26^. Evidence from snow-depth manipulation studies suggests that litter decomposes faster and more carbon and nutrients are released when snow is deeper^27^,^28^,^29^. In the absence of snow cover under a closed canopy, recalcitrant compounds degrade slower and accumulate more in litter tissue^4^,^30^. During the growing season, forest gaps are subject to intensive solar radiation, which decreases soil moisture^31^ and associated microbial activity^32^ and respiration^33^. The consumption of carbon and nutrients in plant residue by soil decomposers could therefore be constrained in forest gaps^23^. Thus, compared with the litter under the undisturbed understory, the litter in forest gaps has a lower potential to be decomposed, but contains more material with refractory components during the growing season. However, numerous studies have indicated that these recalcitrant plant constituents can be polymerized to precursors of stable SOM with the help of specific microorganisms^8^,^34^,^35^. Here, we hypothesize that litter humification is suppressed during the winter but stimulated during the growing season in open forest gaps in these pristine alpine forests due to the contrasting environmental conditions that result from the lack of a forest canopy.

Based on previous studies^4^,^5^, we selected six local types of foliar litter that dominate the alpine forests of the eastern Tibetan Plateau. In this region, studies of winter ecological conditions are important and runoff is primarily generated by snowmelt, which is therefore crucial for the water supply and quality of the upper Yangtze River. Snow cover and freeze-thaw cycles during winter greatly contribute to the release of carbon and nutrients from litter, and previous studies have paved the way for understanding the winter ecological processes in these alpine forests^36^,^37^. In this study, certain optical properties of humification were measured over two years during the winter and growing seasons using a colourimetric method^16^,^17^. The aim of this study is to assess the effects of forest gaps on litter humification and to evaluate whether the effects of forest gap positions on optical properties vary between the winter and growing seasons relative to humus accumulation^5^ and litter decomposition^38^, as described in our previous studies. Our results are expected to broaden the current knowledge regarding the theoretical mechanisms that control carbon and nutrient fluxes from plant litter to soil in these cold biomes.

## Results

### Remaining mass of the six foliar litters

**Figure 1:**
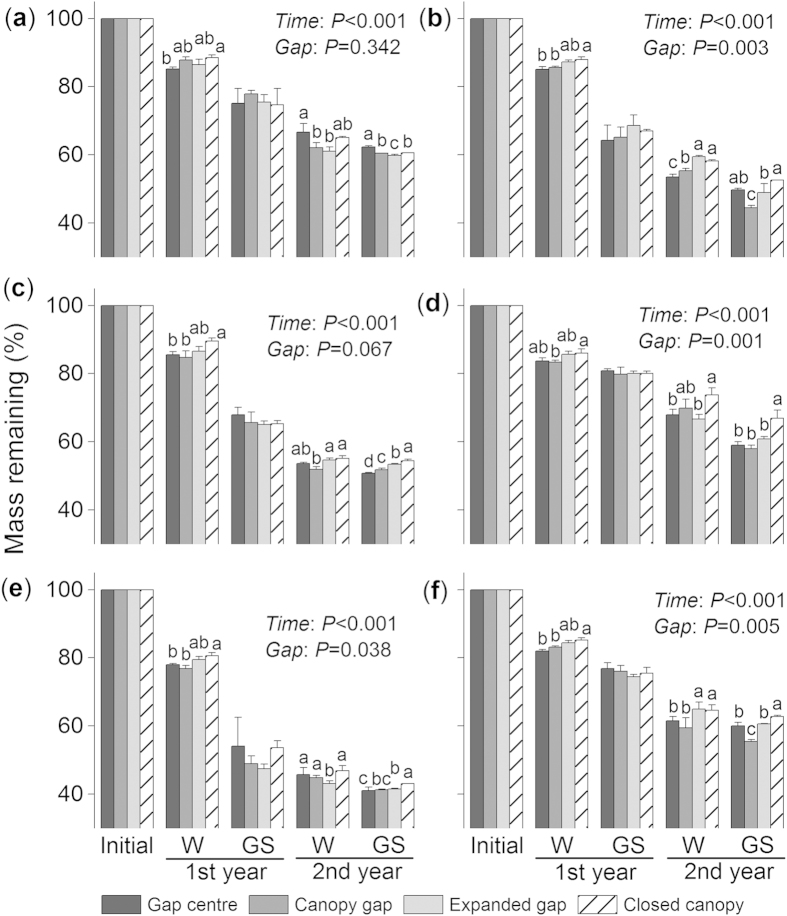
The effects of forest gap positions on the mass remaining of (a)fir, (**b**) cypress, (**c**) larch, (**d**) birch, (**e**) willow and (**f**) azalea foliar litter during the winter (W) and growing seasons (GS) of the two-year humification experiment. Error bars indicate the standard deviations (*n* = 3). *P*-values for incubation time (Time) and gap position treatments (Gap) from repeated measures ANOVA are shown for each species. Values at the same stage with different letters are significantly (*P* < 0.05) different among the four positions.

At the end of the study, the mass of the remaining litter was greater under the closed canopies for all species (Fig. 1b–f), except the fir litter, for which the highest mass remaining was observed in the gap centre (Fig. 1a). Significant differences among the four gap positions were detected during the first winter and during both the winter and growing season of the second year, regardless of the litter species (all *P* < 0.05, Tukey’s HSD; Fig. 1a–f). However, the effect of litter species (*F* = 603.9, *P* < 0.001, repeated measures ANOVA; see Supplementary Table S1 online) was greater than that of gap position (*F* = 24.6, *P* < 0.001) over time. After two years of field incubation, the willow litter exhibited the lowest amount of mass remaining (40% to 43%), followed by (in ascending order) cypress (44% to 53%), larch (51% to 54%), fir (60% to 62%), azalea (55% to 63%), and birch (58% to 67%).

### Forest gaps affect the ΔlogK values of the six foliar litters

**Figure 2:**
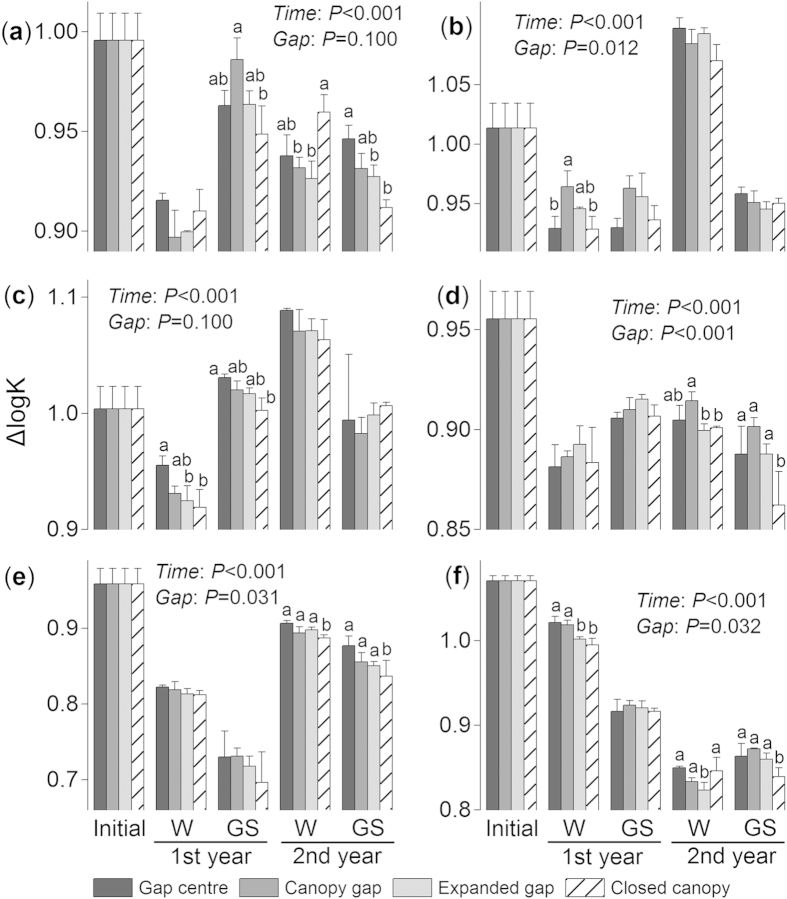
The effects of forest gap positions on the ΔlogK values of (a)fir, (**b**) cypress, (**c**) larch, (**d**) birch, (**e**) willow and (**f**) azalea foliar litter during the winter (W) and growing seasons (GS) of the two-year humification experiment. Error bars indicate the standard deviations (*n* = 3). *P*-values for incubation time (Time) and gap position treatments (Gap) from repeated measures ANOVA are shown for each species. Values at the same stage with different letters are significantly (*P* < 0.05) different among the four position.

The ΔlogK values varied greatly among the different seasons (*F* = 224.4, *P* < 0.001; see Supplementary Table S1 online) and litter species (*F* = 1305.4, *P* < 0.001). During the first winter, considerable decreases in the ΔlogK values were observed for all litter species (Fig. 2a–f). The ΔlogK values of the fir (Fig. 2a), larch (Fig. 2c) and birch (Fig. 2d) litter increased during the following growing season but then decreased during the second growing season. A remarkable increase in the ΔlogK value of the cypress litter was observed during the second winter, but the value declined considerably during the following growing season (Fig. 2b). The ΔlogK value consistently decreased until the first growing season for the willow litter (Fig. 2e) and until the second winter for the azalea litter (Fig. 2f).

**Figure 3:**
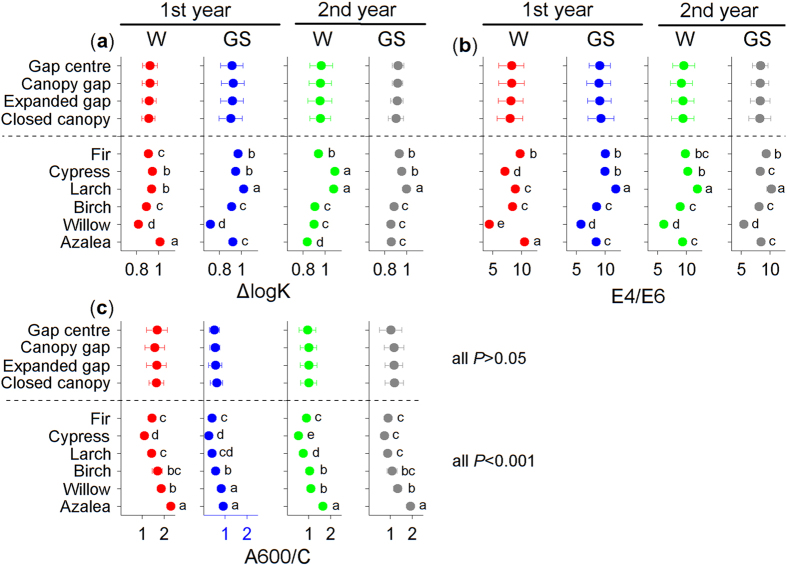
Means and 95% confidence intervals of the (a) ΔlogK, (b) E4/E6 and (c) A600/C values of humification after the first (second) winter and growing season in a given gap position for all litter species combined (above dashed line, all *P* > 0.05) and for the individual litter species for all gap positions (below dashed line, all P < 0.001). Values at the same stage with different letters are significantly (*P* < 0.05) different among the six litters.

Different forest gap positions significantly impacted the ΔlogK value over time (*F* = 16.9, *P* < 0.001, repeated measures ANOVA) and at each separate stage (all *P* < 0.01, two-way ANOVA; Table 1). Overall, lower (*P* < 0.05, Tukey’s HSD) ΔlogK values were observed under the closed canopies than in the gap centres and canopy (expanded) gaps. However, the ΔlogK values of the fir and azalea litter were higher (*P* < 0.05, Tukey’s HSD) under the closed canopies compared with the expanded (canopy) gap during the second winter. When all treatments of a certain species were combined, the willow litter exhibited the lowest ΔlogK value during the first year (*P* < 0.05, Tukey’s HSD; Fig. 3a).

### Forest gaps affect the E4/E6 values of the six foliar litters

**Figure 4:**
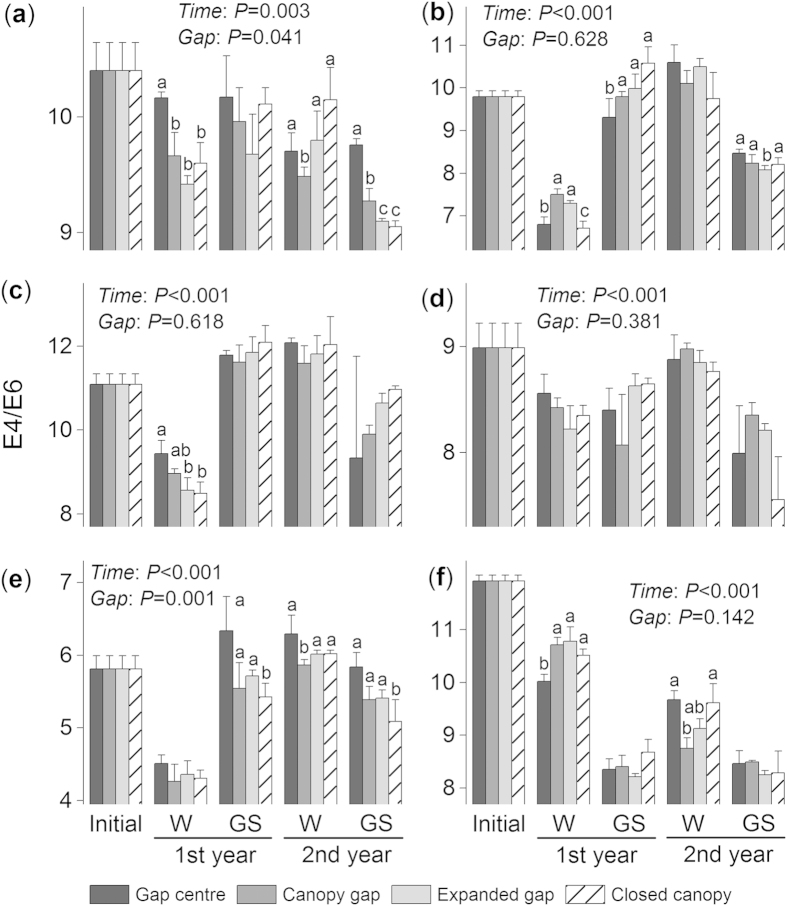
The effects of forest gap positions on the E4/E6 values of (a)fir, (**b**) cypress, (**c**) larch, (**d**) birch, (**e**) willow and (**f**) azalea foliar litter during the winter (W) and growing seasons (GS) of the two-year humification experiment. Error bars indicate the standard deviations (*n* = 3). *P*-values for incubation time (Time) and gap position treatments (Gap) from repeated measures ANOVA are shown for each species. Values at the same stage with different letters are significantly (*P* < 0.05) different among the four positions.

The E4/E6 values varied greatly among the seasons (*F* = 194.5, *P* < 0.001; see Supplementary Table S1 online) and litter species (*F* = 1125.3, *P* < 0.001). Considerable decreases in the E4/E6 values of the six foliar litter types were observed during the first winter (Fig. 4a–f), but the values (except azalea litter) increased during the following growing season and then declined during the second growing season. The E4/E6 value of the azalea litter consistently decreased during the entire first year and then increased during the second winter (Fig. 4f).

Different forest gap positions significantly affected the E4/E6 values across litter species at all stages except the second growing season (*F* = 0.2, *P* = 0.923; Table 1). For the fir (Fig. 4a), larch (Fig. 4c) and willow foliar litter (Fig. 4e), lower (*P* < 0.05, Tukey’s HSD) E4/E6 values were observed under the closed canopies compared with the other positions, except for the fir and willow litter during the second winter. However, the E4/E6 values of the cypress litter during the two growing seasons (Fig. 4b) and those of the azalea litter during the two winter seasons (Fig. 4f) were higher (*P* < 0.05, Tukey’s HSD) under the closed canopies. There were no significant (*P* > 0.05, Tukey’s HSD) differences among the forest gap positions for the birch litter (Fig. 4d). In addition, the willow litter exhibited the lowest (*P* < 0.05, Tukey’s HSD; Fig. 3b) E4/E6 value at all stages when all of the forest gap treatments for a particular species were combined.

### A600/C values and Kumada classification

**Figure 5:**
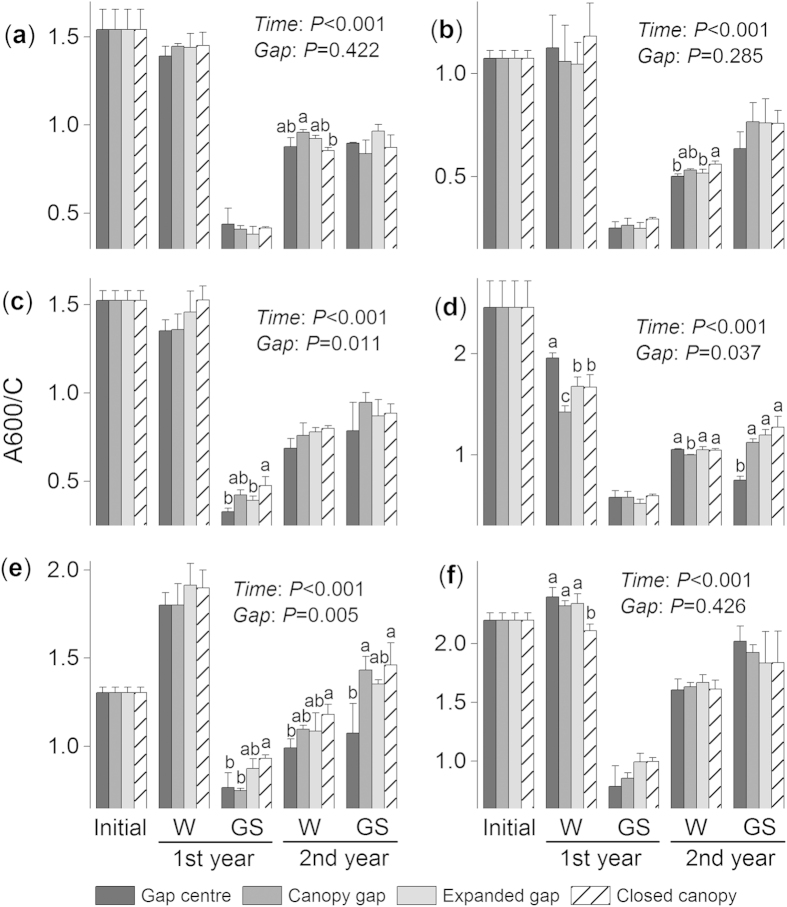
The effects of forest gap positions on the A600/C values of (a) fir, (**b**) cypress, (**c**) larch, (**d**) birch, (**e**) willow and (**f**) azalea foliar litter during the winter (W) and growing seasons (GS) of the two-year humification experiment. Error bars indicate the standard deviations (*n* = 3). *P*-values for incubation time (Time) and gap position treatments (Gap) from repeated measures ANOVA are shown for each species. Values at the same stage with different letters are significantly (*P* < 0.05) different among the four positions.

Consistent with the ΔlogK and E4/E6 values, the A600/C values varied greatly among seasons (*F* = 1983.4, *P* < 0.001; see Supplementary Table S1 online) and showed significant species effects (*F* = 878.3, *P* < 0.001). During the first winter, the litter of the three needle species (fir, cypress and larch) exhibited little changes in the A600/C values (Fig. 5a–c). The broadleaf birch litter (Fig. 5d) decreased but the two shrub litters (willow and azalea; Fig. 5e,f) increased during the first winter. The A600/C values of all litter species decreased considerably during the following growing season and then increased during the second winter (Fig. 5a–f).

**Figure 6:**
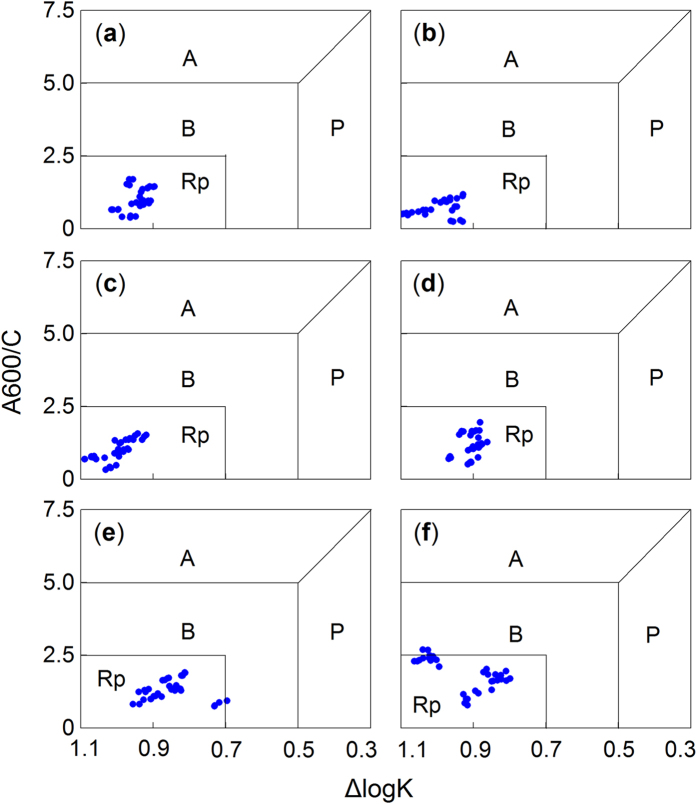
The types of accumulative humic substances in (a) fir, (**b**) cypress, (**c**) larch, (**d**) birch, (**e)** willow and (**f**) azalea foliar litter during the two-year humification experiment based on a modified Kumada classification.

Overall, a significant effect of the gap positions on the A600/C value was detected over time (*F* = 9.0, *P* = 0.032, repeated measures ANOVA; see Supplementary Table S1 online). For the cypress, larch and willow litter, the A600/C values were higher (*P* < 0.05, Tukey’s HSD) under the closed canopies than in the gap centres (or canopy gaps). However, the A600/C values of the fir litter during the second winter (Fig. 5a) and the birch (Fig. 5d) and azalea litter (Fig. 5f) during the first winter were lower (*P* < 0.05, Tukey’s HSD) under the closed canopies. The shrub willow and azalea litter had higher A600/C values than the litter of the other needle species (i.e., fir, cypress and larch) when all treatments of a particular species were combined (Fig. 3c). The accumulated humic substance, which was represented by the A600/C and ΔlogK values, was determined to be type Rp (i.e., young, according to the Kumada classification) for all six litter species (Fig. 6a–f). The two shrub litters, willow and azalea, had more mature (type B) humus (Fig. 6e,f).

## Discussion

Lower ΔlogK or E4/E6 values indirectly indicate a higher degree of humification, whereas the A600/C values exhibit a reverse pattern^16^,^17^. Our results showed that the plant litter was substantially humified during the first winter and the newly formed humus could be decomposed further, suggesting that humus buildup during early humification can be directly controlled by environmental conditions in different seasons as well as by the inherent stability of organic-mineral compounds in the newly accumulated humic substances^34^. This finding is consistent with our previous results^5^. Moreover, the forest gaps significantly affected early litter humification, but these effects varied among seasons and litter species. This observation partially supports our aforementioned hypothesis on the dual responses of litter humification to gap formation between the winter and growing seasons in these alpine forests.

**Figure 7:**
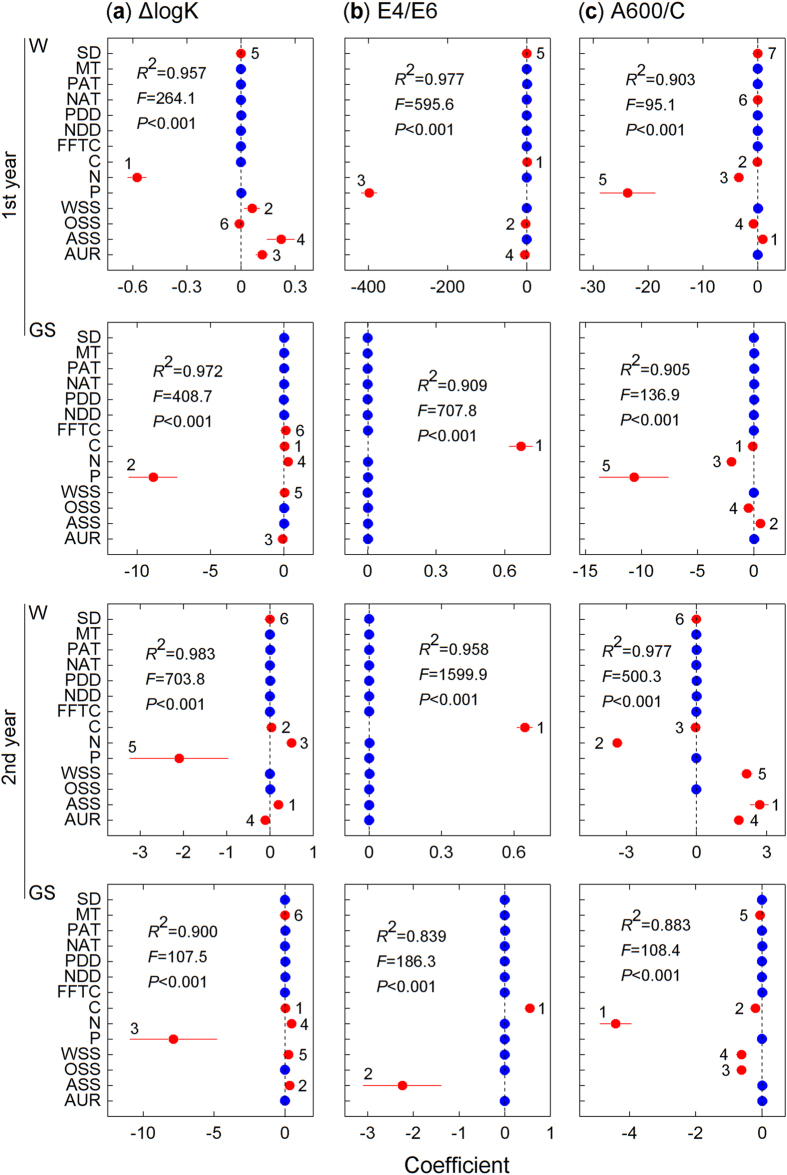
Coefficients and 95% confidence intervals for stepwise analyses of ΔlogK, E4/E6 and A600/C values using seven environmental factors and seven initial chemical compositions during the winter (W) and growing seasons (GS) of the two-year humification experiment. The dominant factors (red) are numbered and the order of priority signifies the step number in the regression model in each panel. SD, snow depth; MT, mean temperature; PAT, positive accumulated temperature; NAT, negative accumulated temperature; PDD, positive degree days; NDD, negative degree days; FFTC, frequency of freeze-thaw cycle; C, carbon; N, nitrogen; P, phosphorus; WSS, water-soluble substances; OSS, organic-soluble substances; ASS, acid-soluble substances; and AUR, acid-unhydrolyzable residues.

Forest gaps have been demonstrated to greatly alter ambient environmental conditions (Table 2) and associated nutrient cycling^23^,^31^,^39^. However, in alpine forests, the lack of canopy cover allows winter snow to accumulate and remain over the litter for long periods (see Supplementary Fig. S1 online), and the thermal resistance provided by the snow cover decreases the risk of freeze-thaw events^26^ (Table 2). Snow depth and duration (from the beginning of accumulation in fall to spring snowmelt) have major implications for subnivean temperature conditions and certain optical properties during litter humification according to our previous observation^4^. Environmental variations caused by alterations in snow depth may contribute to differences in chemical traits^29^ and decomposer activity^28^, suggesting that the thermal resistance provided by snow can enhance litter decomposition during winter^29^. The increased decomposition (Fig. 1; *P* < 0.001) of fresh litter in forest gaps with deep snow cover during the winter was considered to result in the accumulation of fewer resistant compounds (unpublished data) and to negatively correspond to the buildup of humic substances^1^ (Fig. 7a–c). However, the winter snow could not completely offset the microbial injury that results from cold temperatures. The stepwise analysis results showed that the humification process was largely limited by nitrogen and/or phosphorus in these forests (Fig. 7a–c), and the low nitrogen and phosphorus concentrations in the fir and azalea foliar litter (see Supplementary Table S2 online) limited microbial activity in the cold environments, particularly under closed canopies with no snow cover during the winter. This nutrient-limited scenario^7^ may explain the lower degree of humification of the fir and azalea litter, which resulted in higher ΔlogK (Fig. 2a,f) and E4/E6 values (Fig. 4a,f) but lower A600/C values (Fig. 5a,f) under the closed canopies during winter.

At all measurement times, the ΔlogK and E4/E6 values responded positively to the gap position treatment (Table 1; all *P* < 0.05 except the E4/E6 value from the second growing season, two-way ANOVA), suggesting a negative feedback response to litter humification. Our current results imply that the responses of the optical properties of humification to the gap positions resulted in patterns that differed from the accumulation process of humus, which was described in Ni *et al.*^5^. These differences in the effects of forest gaps are attributed to the intricate biochemical pathways of humification and to various monitoring methods because the humification process is complicated and different evaluations may produce contrasting results^9^. Additional studies should be conducted to assess the structural changes associated with humification^14^,^15^. Moreover, large increases in the ΔlogK (Fig. 2a–d) and E4/E6 values (Fig. 4a–e) of certain litter species were observed during the first growing season, which suggested a negative progression of humification. This phenomenon is consistent with our previous results^5^, in which the accumulated humic substances, especially fulvic acid, were young and highly sensitive to temperature^8^ but had low selective preservation with mineral particles^34^. These unstable humic substances could therefore be decomposed during the growing season with a higher temperature relative to the cold winter. Nevertheless, compared with the winter, the humification and decomposition processes of fresh litter during the growing seasons were less sensitive to the forest gap positions, and many variables, such as the mass remaining (Fig. 1a–f) and the E4/E6 (Fig. 4a–f) and A600/C values (Fig. 5a–f), did not vary significantly among the gap positions during some stages. This difference could be attributed to the inherent properties of the forest gap itself. In these alpine forests, the changes in environmental variables, particularly the factors affecting litter humification, such as snow, temperature and related freeze-thaw events (Fig. 7a–c), were greater during the winter than the growing seasons. In addition, this result could be attributed to that fact that our study was initiated during the early winter when large amounts of fresh litter material covered the ground and provided enough labile carbon and nutrients for soil decomposers (manuscript in preparation). These results agree with those of our previous studies within the framework of winter ecological processes in this alpine area^4^,^36^,^37^,^38^.

During the two-year incubation, the decomposition patterns of the six foliar litters were well described by the exponential model^40^ (all *R*^2^ > 0.8; see Supplementary Table S3 and Fig. S2 online), whereas humification did not follow a predictable pattern. Overall, the newly formed humus (type Rp, as classified by the A600/C and ΔlogK values) from most litter species was young based on the Kumada classification^16^, which was slightly modified by Ikeya and Watanabe^17^. The litter of the two shrub species, willow and azalea, exhibited a more mature humus (type B; Fig. 6e,f)^16^. The relatively eutrophic willow litter has higher concentrations of nitrogen and water-soluble substances^5^, and lower C/N and gravimetric acid-unhydrolysable residue (AUR)/N ratios (see Supplementary Table S2 online). Although the willow litter exhibited a higher degree of humification with lower ΔlogK (Fig. 3a) and E4/E6 values (Fig. 3b), this evidence was not sufficient to support the microbial-derived theory^7^. Conversely, the azalea litter exhibited higher C/N and AUR/N values (see Supplementary Table S2 online) and resulted in a high degree of humification with the highest A600/C value, regardless of gap position (Fig. 3c). Moreover, the azalea litter exhibited a continuous humification process with humus accumulation until the end of the experiment (Figs 2f and 4f). This result may be related to the high C/N and AUR/N ratios of the azalea litter; therefore, these recalcitrant materials could be polymerized to precursors of humic substances^34^,^35^ as detected in our study. Although seven environmental factors and seven initial chemical compositions were included in the stepwise analysis, the results ultimately demonstrated that the initial concentrations of carbon and acid-soluble substances (cellulose and hemicellulose) explained most of the total variability in the three variables of humification (Fig. 7a–c). However, the labile components, such as nitrogen and phosphorus, explained less of the total variation but contributed large negative coefficients in the regression model (Fig. 7a–c). These results implied that litter humification in these alpine forests may be plant derived and controlled by recalcitrant materials^1^,^34^ because this process seems to be nutrient (nitrogen and phosphorus) limited. However, it is not yet possible to exclude the metabolic contributions of microorganisms^7^. Previous evidence suggested that the SOM polymerized by resistant compounds has low carbon stability and high temperature sensitivity^8^ because of its low mineral association and selective stabilization^41^. These characteristics can explain why the newly accumulated humic substances were degraded, which was described in our previous work^5^, and clarifies why large increases in the ΔlogK (Fig. 2a–d) and E4/E6 values (Fig. 4a–e) occurred during the first growing season as described above. The six types of foliar litter manipulated in this study were conducted in a 130-year-old mature fir forest, and all five of the other species were dominant at the sampling site (see Methods section). However, this site condition could increase the “home-field” advantage^42^ relative to the other five species. Consequently, the fir foliar litter could be decomposed faster under the original canopy (Fig. 1a) in contrast with the other species.

In a given ecosystem, litter quality is considered the most important and direct factor that regulates decomposition^3^,^43^. Litter chemistry is also important at the global scale^44^. Vascular plant functional types have repeatedly been shown to decompose at distinct rates because of their specific substrate qualities^45^. Throughout the entire experiment, interspecific differences were much larger than the forest gap-induced differences^46^ (see Supplementary Table S1 online). The foliar litters of the willow and azalea shrub species exhibited greater degrees of humification, with lower ΔlogK (Fig. 3a) and E4/E6 values (Fig. 3b) and higher A600/C values (Fig. 3c) than those of the tree species. These results revealed that the foliar litter from the shrubs could be humified more rapidly than the tree litter in these cold biomes. There is increasing evidence that alpine shrubs have undergone rapid expansion and upward displacement related to climate warming in recent decades^47^. Consequently, in these undisturbed alpine ecosystems, expanded shrubs may enhance soil carbon sequestration in the future. Although the release of carbon from the soil may be stimulated due to climate warming^48^, shrub species have become widespread as the temperature has increased and therefore more newly shed litter materials can be transformed into stable SOM^49^ without decomposing or leaching carbon to other systems. Hence, litter from dwarf shrubs should receive more attention in studies on the climate-carbon feedback cycle in regions with high latitudes and altitudes.

In summary, during the two years of field incubation, high degrees of humification of newly shed litter were observed in the alpine forests; however, the newly accumulated humic substances were still young and had low mineral stability, which suggests that the early humification process in these cold biomes may be plant derived due to the high occurrence of recalcitrant compounds. The shrub litter was humified more rapidly than the needle litter, which suggests that the predicted shrub expansion caused by climate warming could promote regional carbon storage. Moreover, forest gap-induced canopy openings significantly affected the early humification process of the plant litter during the winter and growing seasons, but these effects varied among the sampling seasons and litter species. Our results suggest that gap formation during forest regeneration hampers the conversion of plant residues to stable soil organic matter and reduces the security of long-term soil fertility in alpine forests.

## Methods

### Site description

This study was conducted at the Long-term Research Station of Alpine Forest Ecosystems, which is located in the eastern Tibetan Plateau, China (31°14′ N, 102°53′ E and 3579 to 3582 m *a.s.l.*). The annual mean temperature and precipitation are 2.7 °C and 850 mm, respectively. Snow begins to accumulate in late October and melts in late April of the following year with the maximum snow depth reaching approximately 50 cm. Fir (*Abies faxoniana*) is the constructive species in the study area, and the forest is dominated by coniferous cypress (*Sabina saltuaria*) and deciduous larch (*Larix mastersiana*) and birch (*Betula albo-sinensis*). The main understory shrubs include willow (*Salix paraplesia*) and azalea (*Rhododendron lapponicum*). Canopy gap (a “hole” in the plant canopy below 2 m) and expanded gap (canopy gap plus the area that expands to the bases of the surrounding canopy trees^20^) cover 13% and 23%, respectively, of the experimental site^50^. The soil is classified as a Cambisol (World Reference Base taxonomy^51^). The depth of the fresh litter layer is 7 ± 1 cm, and its carbon storage is 1.6 ± 0.6 t C ha^−1^. The carbon, nitrogen, phosphorous and humus contents in the organic layer are 160 ± 16, 58 ± 1, 1.70 ± 0.01 and 61 ± 6 mg g^−1^, respectively^5^.

### Experimental design

The experimental site is located in a 130-year-old evergreen fir forest. Three similar oval forest gaps (ca. 20 m × 25 m in size) with homogeneous gap formation patterns (tree fall) and durations (ca. 20 years) were established as replicates. A randomized complete factorial design was used, with gap position as the main factor nested by a species factor. Four treatments were considered in various gap positions along a gradient, the gap centre, canopy gap, expanded gap^20^,^38^, and closed canopy (control, around the fir trunk). Six dominant types of foliar litter (fir, cypress, larch, birch, willow and azalea) were studied in each position separately.

The six senesced foliar litters mentioned above were collected from the ground at the site in October 2012 to coincide with natural foliage death. Air-dried litter samples (10 ± 0.05 g each) were placed in 20 cm × 25 cm nylon litterbags (with mesh sizes of 1.0 mm on the top and 0.5 mm on the bottom) and transferred to the four gap positions from the gap centre to the closed canopy on 15 November 2012. Two litterbags of each species were randomly harvested from each treatment on 24 April 2013, 30 October 2013, 24 April 2014 and 29 October 2014 after 159, 348, 524 and 712 days, respectively. These sampling dates denote the approximate ends of the winter and growing seasons at the experimental site according to our previous observations^4^,^5^,^36^,^37^,^38^. Litter temperatures were recorded every 2 hours using data loggers (iButton DS1923-F5, Maxim/Dallas Semiconductor, Sunnyvale, CA, USA) (Table 2 and see Supplementary Fig. S1 online), which were placed in the litterbags located at each gap position. Heavy snowfall and earthquakes present sampling challenges in these alpine forests; therefore, the snow depths were manually measured in triplicate on the sampling dates (see Supplementary Fig. S1 online).

### Sample analyses

Humic substances usually show a featureless absorption curve in which the optical density increases with decreasing visible wavelength^16^,^17^. Because the ΔlogK and E4/E6 (465 and 665 nm) values decrease as humus conjugates, lower values signify higher degrees of humification. Air-dried subsamples were extracted in 0.1 moles/l sodium hydroxide and sodium pyrophosphate. The filtered solution of alkali-extractable humic substances was dissolved in 0.05 moles/l sodium bicarbonate^52^ and then analysed using a spectrometer (TU-1901, Puxi, Beijing, China).

### Data calculations and statistical analyses

The values of ΔlogK, E4/E6 and A600/C (absorbance at 600 mm per mg C per ml extraction) were calculated as follows^17^:


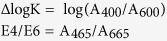


where A_400_, A_600_, A_465_ and A_665_ are the absorbances measured at 400, 600, 465 and 665 nm, respectively. In addition, for A600/C, C represents the mg carbon per ml extraction.

We calculated the mean temperature, positive and negative accumulated temperature, positive and negative degree days (i.e., number of days that the daily mean temperature was above or below 0 °C), and frequency of freeze-thaw cycle (the quotient of the number of freeze-thaw cycle and the days of a certain stage; Table 2) for the various stages and for each treatment. One freeze-thaw cycle was completed when the threshold of 0 °C was crossed twice for at least 3 hours^53^. No significant ‘site effect’ was observed for any of the measured variables according to one-way analysis of variance (ANOVA) when site was used as a fixed factor. A multivariate ANOVA was performed to determine the effects of litter species and gap position treatments on the remaining mass, ΔlogK, E4/E6 and A600/C values over time. The responses of the six individual litter species were also tested by repeated measures ANOVA. At each stage, a two-way ANOVA was performed to test the effects of litter species and gap position treatments on the ΔlogK, E4/E6 and A600/C values. A one-way ANOVA with multiple comparisons using Tukey’s honestly significant difference (HSD) post-hoc test was conducted to test for significant differences among the four treatments at *P* = 0.05 level. The homogeneity of variance was determined before conducting ANOVAs, and the data were logarithmically transformed when required. Each pair of the four treatments (total 6 pairs) for the variable mass remaining was evaluated using a pairwise t test, and only the pairs that exhibited significant differences at *P* = 0.05 were exponentially fit. A stepwise regression analysis was used to detect the dominant factor(s) controlling the ΔlogK, E4/E6 and A600/C values at each stage, and the variables were included in the regression model at the *F* < 0.05 level. In addition, to determine the effects of gap position treatments and litter species on the ΔlogK, E4/E6 and A600/C values, reasonable combinations of these variables were assessed. ANOVAs and stepwise analyses were performed using MATLAB R2012a (MathWorks Inc., Natick, MA, USA), and locally weighted exponential fitting was conducted using Origin Pro9.0 (OriginLab, Northampton, MA, USA).

## Additional Information

**How to cite this article**: Ni, X. *et al.* Forest gaps slow the sequestration of soil organic matter: a humification experiment with six foliar litters in an alpine forest. *Sci. Rep.* **6**, 19744; doi: 10.1038/srep19744 (2016).

## Supplementary Material

Supplementary Information

Supplementary Dataset 1

Supplementary Dataset 2

Supplementary Dataset 3

Supplementary Dataset 4

Supplementary Dataset 5

Supplementary Dataset 6

## Figures and Tables

**Table 1:** Results of two-way ANOVA of the effects of litter species (Species), gap position treatments (Gap) and their interactions on the ΔlogK, E4/E6 and A600/C values at each stage.

Stage	Species		Gap		Species × Gap	
	*F*value	*P*value	*F*value	*P*value	*F*value	*P*value
ΔlogK						
1st winter	563.5	<0.001	7.2	<0.001	4.3	<0.001
1st growing season	630.3	<0.001	6.9	<0.001	1.4	0.211
2nd winter	1450.9	<0.001	6.3	0.001	4.0	<0.001
2nd growing season	162.7	<0.001	5.6	0.002	1.7	0.090
E4/E6						
1st winter	1801.7	<0.001	8.7	<0.001	9.2	<0.001
1st growing season	421.7	<0.001	3.3	0.028	2.5	0.008
2nd winter	524.8	<0.001	6.0	0.001	2.4	0.012
2nd growing season	108.2	<0.001	0.2	0.923	1.9	0.053
A600/C						
1st winter	211.0	<0.001	3.6	0.200	4.3	<0.001
1st growing season	245.3	<0.001	9.1	<0.001	2.9	0.003
2nd winter	776.4	<0.001	5.6	0.002	2.4	0.013
2nd growing season	116.9	<0.001	7.3	<0.001	3.3	0.001

**Table 2:** Mean temperature (MT), positive and negative accumulated temperature (PAT and NAT, respectively), positive and negative degree days (PDD and NDD, respectively), and frequency of freeze-thaw cycle (FFTC) in the four gap positions during the two-year humification experiment.

Forest gap positions	MT (°C)	PAT (°C)	NAT (°C)	PDD (°C)	NDD (°C)	FFTC (time d^−1^)
1st winter						
Gap centre	−1.70 (0.30)	106 (20)	−407 (40)	44 (7)	113 (6)	0.435 (0.280)
Canopy gap	−1.70 (0.37)	78 (11)	−375 (58)	33 (9)	121 (13)	0.487 (0.159)
Expanded gap	−1.73 (0.22)	111 (18)	−439 (33)	45 (7)	110 (3)	0.532 (0.054)
Closed canopy	−2.14 (0.19)	104 (35)	−479 (20)	46 (13)	112 (14)	0.564 (0.072)
1st growing season						
Gap centre	8.90 (0.53)^a^	1683 (100)^a^	0 (1)	187 (4)	2 (3)	0.083 (0.017)
Canopy gap	7.66 (0.14)^b^	1451 (27)^b^	−2 (2)	176 (4)	7 (4)	0.095 (0.014)
Expanded gap	7.87 (0.34)^b^	1493 (59)^b^	−5 (4)	183 (6)	6 (6)	0.092 (0.043)
Closed canopy	7.70 (0.14)^b^	1458 (23)^b^	−3 (3)	181 (4)	6 (4)	0.044 (0.016)
2nd winter						
Gap centre	−2.08 (0.63)	136 (20)	−598 (107)	38 (10)	128 (13)	0.342 (0.082)^b^
Canopy gap	−1.88 (0.20)	165 (13)	−592 (33)	43 (4)	132 (4)	0.615 (0.054)^a^
Expanded gap	−1.90 (0.75)	147 (44)	−563 (100)	49 (19)	126 (18)	0.607 (0.059)^a^
Closed canopy	−2.50 (0.22)	118 (8)	−655 (38)	37 (3)	137 (4)	0.484 (0.055)^ab^
2nd growing season						
Gap centre	9.49 (0.65)^a^	1775 (122)^a^	−1 (1)	181 (1)	4 (1)	0.110 (0.063)
Canopy gap	7.75 (0.58)^b^	1452 (108)^b^	−2 (2)	178 (9)	6 (5)	0.080 (0.032)
Expanded gap	7.38 (0.40)^b^	1383 (79)^b^	−2 (3)	184 (3)	3 (3)	0.078 (0.030)
Closed canopy	7.36 (0.38)^b^	1379 (68)^b^	−2 (3)	181 (6)	4 (6)	0.071 (0.011)
2 years						
Gap centre	3.65 (0.50)^a^	3700 (240)^a^	−1007 (126)	450 (19)	247 (21)	0.242 (0.095)
Canopy gap	2.96 (0.15)^ab^	3145 (86)^b^	−972 (45)	430 (11)	266 (14)	0.319 (0.021)
Expanded gap	2.91 (0.25)^ab^	3135 (62)^b^	−1010 (136)	460 (34)	245 (29)	0.327 (0.017)
Closed canopy	2.61 (0.11)^b^	3058 (57)^b^	−1139 (29)	445 (17)	259 (12)	0.291 (0.027)

Values are means of *n* = 3 observations, with standard deviations shown in parentheses. Values in the same columns with different superscript letters are significantly (*P* < 0.05) different among the four gap positions at each stage based on multiple comparison with Tukey’s HSD.
