# Supplementary material for: Forest gaps slow the sequestration of soil organic matter: a humification experiment with six foliar litters in an alpine forest
Source: Sci Rep. 2016 Jan 21;6:19744. doi: 10.1038/srep19744 (PMC4726255; doi:10.1038/srep19744)
Supplement: Supplementary Information [file srep19744-s1.pdf]

Supplementary information for

Forest gaps slow the sequestration of soil organic matter: a humification experiment with six foliar litters in an alpine forest

Xiangyin Ni, Wanqin Yang, Bo Tan, Han Li, Jie He, Liya Xu & Fuzhong Wu*

Long-term Research Station of Alpine Forest Ecosystems, Key Laboratory of Ecological Forestry Engineering, Institute of Ecology and Forestry, Sichuan Agricultural University, Chengdu 611130, China

* Corresponding author (E-mail address: wufzchina@163.com)

Tel.: 86-28-86290957; fax: 86-28-86290957

Table S1 Repeated measures ANOVA results of the incubation time (Time), litter species (Species), gap position treatments (Gap) and their interactions on mass remaining, ΔlogK, E4/E6 and A600/C values over time.

Source of variation		Mass remaining		ΔlogK		E4/E6		A600/C	
	df	*F* value	*P* value	*F* value	*P* value	*F* value	*P* value	*F* value	*P* value
Time	3	4894.1	<0.001	224.4	<0.001	194.5	<0.001	1983.4	<0.001
Species	5	603.9	<0.001	1305.4	<0.001	1125.3	<0.001	878.3	<0.001
Gap	3	24.6	<0.001	16.9	<0.001	2.0	0.123	9.0	0.032
Time × Species	15	75.2	<0.001	291.1	<0.001	73.6	<0.001	19.1	<0.001
Time × Gap	9	5.8	0.001	2.6	0.010	2.5	0.027	5.1	<0.001
Species × Gap	15	2.9	0.003	2.3	0.014	2.0	0.034	3.4	0.001
Time × Species × Gap	45	3.1	<0.001	2.2	<0.001	2.7	<0.001	3.5	<0.001

Table S2 The *P*-values of the pairwise t tests before conducting locally weighted exponential fitting of mass remaining with humified days.

Species	GC-CG	GC-EG	GC-CC	CG-EG	CG-CC	EG-CC
Fir	0.806	0.021	0.751	0.005	0.608	0.001
Cypress	0.490	0.001	<0.001	<0.001	<0.001	0.853
Larch	0.003	0.179	0.077	<0.001	<0.001	0.230
Birch	0.380	0.148	<0.001	0.705	<0.001	<0.001
Willow	0.051	0.083	0.007	0.850	<0.001	<0.001
Azalea	0.002	0.181	0.003	0.003	<0.001	0.050

GC, gap center; CG, canopy gap; EG, expanded gap; CC, closed canopy.

Table S3 The initial concentrations of some chemical compositions of the six types of foliar litter. The results were published in Ni et al. (2015).

Species	C (%)	N (%)	P (%)	WSS (%)	OSS (%)	ASS (%)	AUR (%)	C:N quotient	AUR:N quotient
Fir	50.56 (2.96)[a]	0.88 (0.003)[c]	0.11 (0.010)[b]	40.83 (0.54)[ab]	27.62 (2.28)[ab]	27.36 (1.33)[b]	23.92 (2.54)[b]	57.77 (3.53)[b]	27.33 (2.92)[b]
Cypress	51.64 (1.77)[a]	0.88 (0.010)[c]	0.12(0.006)[ab]	35.74 (0.69)[c]	33.16 (3.43)[a]	32.43 (1.29)[a]	20.60 (3.41)[b]	58.86 (2.21)[b]	23.48 (3.89)[b]
Larch	54.35 (0.63)[a]	0.86 (0.041)[c]	0.13 (0.002)[a]	40.08 (1.08)[b]	19.11 (0.68)[c]	29.24 (0.87)[ab]	21.46 (0.94)[b]	63.32 (3.49)[b]	25.01 (2.04)[b]
Birch	49.69 (1.45)[ab]	1.33 (0.022)[a]	0.09 (0.004)[c]	25.06 (1.96)[d]	11.43 (0.75)[d]	27.74 (0.94)[b]	50.96 (0.96)[a]	37.24 (1.35)[c]	38.19 (1.01)[a]
Willow	45.23 (1.65)[b]	1.15 (0.028)[b]	0.11 (0.002)[b]	41.71 (0.32)[ab]	18.48 (1.57)[c]	28.56 (1.88)[b]	26.15 (3.29)[b]	39.49 (2.18)[c]	22.79 (2.45)[b]
Azalea	50.29 (1.60)[a]	0.67 (0.020)[d]	0.11(0.009)[bc]	43.14 (1.16)[a]	25.84 (2.29)[b]	27.00 (0.59)[b]	21.84 (3.42)[b]	75.54 (4.47)[a]	32.90 (6.13)[ab]

Values are means of n=3 observations, with standard deviations shown in parentheses. Values in the same columns with different superscript letters are significantly (P<0.05) different among the six litter species based on multiple comparisons using Tukey's HSD. C: carbon, N: nitrogen, P: phosphorus, WSS: water-soluble substances, OSS: organic-soluble substances, ASS: acid-soluble substances, AUR: acid-unhydrolytic residues.

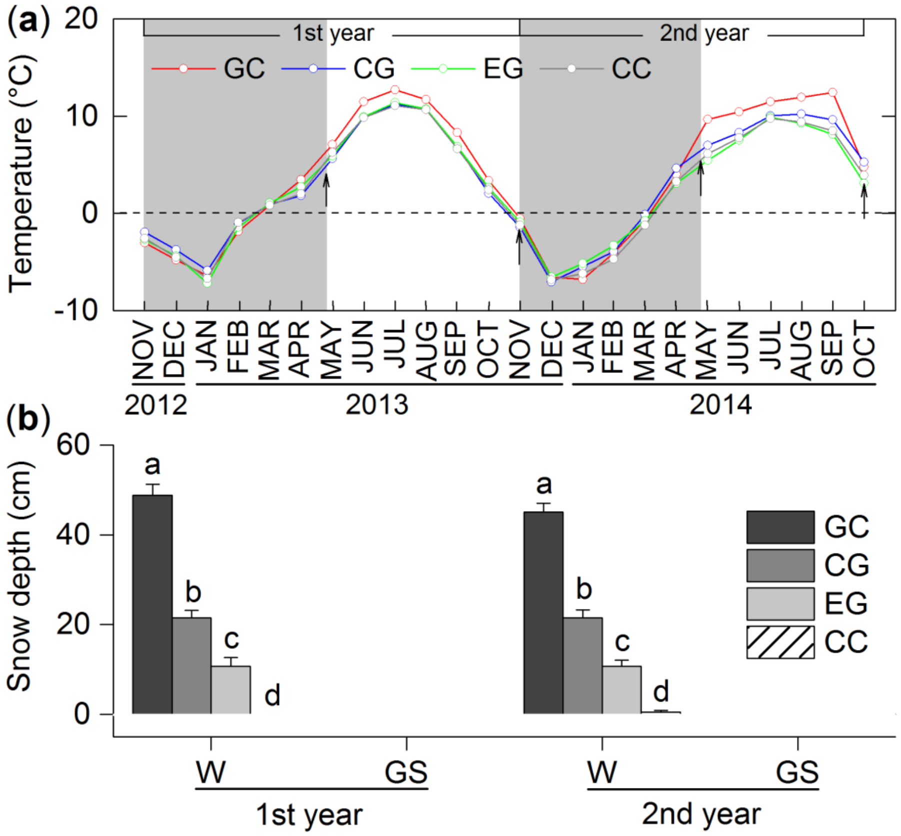

Figure S1 (a) Monthly temperatures and (b) snow depths in the four gap positions during the winter (W) and growing seasons (GS) of the two-year humification experiment. The winter months are shown as grey-shaded areas and the sampling dates are marked by arrows in panel (a). Error bars present standard deviations (n=9) for panel (b). GC, gap center; CG, canopy gap; EG, expanded gap; CC, closed canopy.

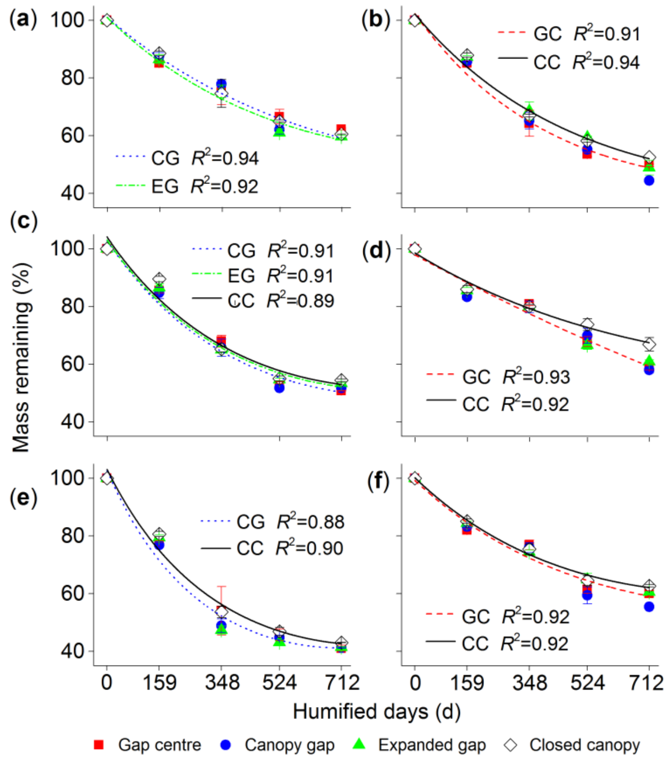

Figure S2 Locally weighted exponential fitting of mass remaining of (a) fir, (b) cypress, (c) larch, (d) birch, (e) willow and (f) azalea foliar litter with humified days. Differences in each pair of the four gap position treatments throughout the entire incubation time were examined using pairwise t tests at a P=0.05 level of significance. Only pairs with significant differences (from Table S2) were fit exponentially, and the R^2 values are shown.
